# Psychosocial Correlates of Mental Health and Well-Being During the COVID-19: The Spanish Case

**DOI:** 10.3389/fpsyg.2020.609815

**Published:** 2020-11-25

**Authors:** Sara Esteban-Gonzalo, Juan Luis González-Pascual, María Caballero-Galilea, Laura Esteban-Gonzalo

**Affiliations:** ^1^Faculty of Biomedicine, Department of Psychology, Universidad Europea de Madrid, Madrid, Spain; ^2^Faculty of Biomedicine, Department of Nursing, Universidad Europea de Madrid, Madrid, Spain; ^3^Faculty of Medicine, Department of Nursing, Universidad Autónoma de Madrid, Madrid, Spain

**Keywords:** wellbeing, mental health, COVID-19, Spain, pandemic

## Abstract

**Background:**

The COVID-19 pandemic has hit almost all countries around the globe, seriously affecting the welfare of populations. Spain is especially hard-hit. In this context, the purpose of the present study is to analyze social, demographic, and economic correlates of mental health during the COVID-19 pandemic in the population residing in Spain.

**Method:**

The sample of this cross-sectional study was comprised of 801 participants aged 18 or older and residing in Spain. Data collection was carried out during March and April 2020. Data of mental health (GHQ12) and well-being (Positive and Negative Affect Schedule) indicators, and those of a wide number of social, demographic, and economic variables were recorded. Linear regression models were built to value associations between mental health and social, demographic, and economic indicators.

**Results:**

Mental health morbidity was higher in women, younger people, individuals with medium studies, people with fewer children, singles, students, and unemployed individuals. Positive affect was higher among women, people with a high level of studies, those not co-living with dependent seniors, the self-employed, the employed, and those working outside home. Negative affect was negatively associated with age and number of children and was higher among women, people with basic studies, singles, individuals co-living with dependent seniors, homemakers, and students.

**Conclusion:**

The most vulnerable populations were found to be women, younger people, people with basic or medium studies, students and individuals with no remunerated activities, single populations, and those co-living with dependent seniors as well as those with a reduced number of children.

## Introduction

The entire world is now struggling to overcome one of the most devastating pandemics of the XXI century, until now (Remuzzi and Remuzzi, 2020; World Health Organization [WHO], 2020b; Zu et al., 2020). COVID-19 has hit almost all countries around the globe generating important consequences at different levels. Economic, social, and public health systems have been seriously overwhelmed by the pandemic, putting the welfare state at great risk (Alvarez et al., 2020; Dong et al., 2020; Figari and Fiorio, 2020; Nwogugu, 2020). Particularly in Spain between March 19th and April 26th, 2020, there were 212,640 new detected infections and 22,329 deaths (Estadística, 2019). Experts from many disciplines—epidemiologists, economists, and politicians—are confronting this threat by collectively analyzing how the virus behaves and thereby implementing a great variety of changes in our societies (Atkeson, 2020; Ferguson et al., 2020; Fernandes, 2020).

In order to inhibit the spread of the virus, most countries have established some form of a state of emergency including quarantine periods in which citizens are under strict lockdown and isolation (Parmet and Sinha, 2020; World Health Organization [WHO], 2020a). While this measure has been found to be effective in controlling the progress of the virus (Nussbaumer-Streit et al., 2020), such aggressive restrictions have seriously impacted society as a whole with significant consequences for psychological, social, and economic welfare (Chatterjee et al., 2020; Ho et al., 2020; Lewnard and Lo, 2020). In a context in which education centers, shops and businesses are closed, and most economic activities have been canceled, the social drama has reached incalculable limits (Anderson et al., 2020; Singh and Adhikari, 2020).

In periods of uncertainty such as natural disasters, economic crises, and serious health threats, a great variety of studies have found significant changes in people’s mental health and well-being (Pollard, 2001; Kramer and Bala, 2004; Shannon and Lee, 2008; Afifi et al., 2012). The existing studies in Spain on mental health during COVID-19 have found higher prevalence of psychological distress in women and people of lower middle age. Work situation, living with children under 16, and presence of symptoms of the virus were also found to be predictors of mental health (Gómez-Salgado et al., 2020). Others studies carried out in Spanish population have analyzed the information received, prevention measures, beliefs, concerns, and population’s knowledge about COVID-19, concluding that the degree of concern for COVID-19 and the number of hours spent consulting information on COVID-19 had predictive effects on psychological health (Domínguez-Salas et al., 2020). Also, similar studies have pointed out that being in the older age group, economic stability, and the belief that adequate information had been provided about the pandemic were negatively related to psychological distress. Nevertheless, having symptoms associated with the virus or to have a close relative infected was associated with symptoms of depression, anxiety, or posttraumatic stress disorder (González-Sanguino et al., 2020). Conducting leisure activities and the perception of being in good health have also been found associated with a better mental health (Rodríguez-Rey et al., 2020a).

Similar studies carried out in United Kingdom have reported higher self-harm behaviors and thoughts of suicide among people experiencing socioeconomic disadvantage, unemployment, disability, chronic physical illnesses, mental disorders, and COVID-19 diagnosis (Iob et al., 2020). Preexisting physical and mental health conditions and low social support were also associated with depressive symptoms (Frank et al., 2020). Complementary studies in Italy, a country similarly affected by the pandemic, showed that those with a family member infected by COVID-19 and young people who had to work outside home presented higher levels of anxiety and stress (Mazza et al., 2020). These studies have also emphasized the risk of psychological distress among parents due to school closures and suspended educational services for children (Fontanesi et al., 2020).

Most existing long-term studies on global pandemics were carried out in China and other Asian countries during the SARS pandemic or during the Ebola and influenza pandemics (Brooks et al., 2020). According to these studies, those who were quarantined reported high prevalence of psychological distress and disorders. General psychological symptoms, emotional disturbance, depression, stress, low mood, irritability, posttraumatic stress symptoms, and emotional exhaustion were found among those affected by quarantine (Person et al., 2004; Mihashi et al., 2009; Yoon et al., 2016; Brooks et al., 2018). People in quarantine after being in contact with those who potentially had SARS reported fear, nervousness, sadness, or guilt (Reynolds et al., 2008). The few studies on sleep disorders during COVID-19 have found higher prevalence of poor sleep quality among health workers when compared with other professions (Huang and Zhao, 2020) and quality of sleep being positively associated with social support (Xiao et al., 2020b) and social capital (Xiao et al., 2020a) and negatively associated with levels of stress and anxiety (Xiao et al., 2020b). It has also been found that four to six months after quarantine, anxiety and feelings of anger decreased (Jeong et al., 2016). However, some long-term effects of quarantine such as alcohol use and dependency symptoms persisted even after three years among sanitary workers (Wu et al., 2008), as did avoidance behaviors such as minimized contact with others and staying clear of crowded enclosed places and public spaces (Reynolds et al., 2008).

The impact of a pandemic on mental health does not seem to affect everyone at the same level. A study carried out in Australia during the 2007 influenza pandemic found that younger age (Pollard, 2001; Kramer and Bala, 2004; Shannon and Lee, 2008; Afifi et al., 2012; Anderson et al., 2020; Chatterjee et al., 2020; Gómez-Salgado et al., 2020; Lewnard and Lo, 2020; Singh and Adhikari, 2020), lower educational status, female gender, and having kids could exacerbate this impact (Taylor et al., 2008). Stressors during quarantine should also be considered. The duration of quarantine seems to be associated with posttraumatic stress symptoms, avoidance behaviors, and anger (Hawryluck et al., 2004; Pellecchia et al., 2015). Fears of infection have also been associated with psychological outcomes even several months later (Jeong et al., 2016). Confinement, loss of usual routine, and reduced social and physical contact have been associated with boredom and frustration, generating distress among quarantined individuals (Blendon et al., 2004; Robertson et al., 2004; Cava et al., 2005; Reynolds et al., 2008; Braunack-Mayer et al., 2013). Difficulties in taking part in day-to-day activities, shopping for basic needs, or participating in social networking could enhance this frustration (Hawryluck et al., 2004; Jeong et al., 2016). Inadequate supplies and poor information have also been found to be associated with frustration, anxiety, anger, confusion, and stress (Blendon et al., 2004; Reynolds et al., 2008; Pellecchia et al., 2015; Jeong et al., 2016).

Lastly, post-quarantine effects may also be taken into account. Both the economy and individuals—particularly the most vulnerable—suffer from the impact of financial loss when people are unable to work. Considerable socioeconomic distress and symptoms of psychological disorders may materialize (Mihashi et al., 2009; Pellecchia et al., 2015; Jeong et al., 2016). Social stigma and rejection after quarantine were reported among those more exposed to the pandemic such as health workers who suffered from social discrimination, fear, and suspicion (DiGiovanni et al., 2004; Hawryluck et al., 2004; Cava et al., 2005; Lee et al., 2005).

Mental health is defined by the World Health Organization as “a state of well-being in which the individual realizes his or her own abilities, can cope with the normal stresses of life, can work productively and fruitfully, and is able to make a contribution to his or her community” (World Health Organization [WHO], Department Whosa, Health WHODoM, and Abuse, 2004). Mental health can be measured by different diagnostic methods such as the Composite International Diagnostic Interview (CIDI) or Schedules for Clinical Assessment in Neuropsychiatry (SCAN). However, short questionnaires have been found to be useful and valid measures of mental health to facilitate a general picture of the mental health status of an individual or a population and identify risk groups or monitor changes over time (Hoeymans et al., 2004).

Well-being is a key aspect of mental health (Galderisi et al., 2015). The hedonic well-being approach defines well-being in terms of pleasure and pain (Ryan and Deci, 2001), considering feelings such as happiness, sadness, anger, stress, and pain. It is commonly measured by analyzing positive and negative experiences in people’s daily lives with experience sampling methodologies (ESM) or similar methods based on diary techniques to appraise subjective experiences in daily life such as the Day Reconstruction Method (Diener et al., 1985b; Keyes et al., 2002; Kahneman et al., 2004; Steptoe et al., 2015). Empirical findings suggest that positive and negative affect should be separately measured as independent dimensions by asking people about their feelings at a given period of time (Diener et al., 1985a).

For all these reasons, the purpose of the present study is to analyze social, demographic, and economic correlates of mental health during the COVID-19 pandemic in the population residing in Spain. We aim to evince the factors capable of predicting improvement or exacerbation of psychological distress.

## Method

### Study Design and Participants

This cross-sectional study was designed to assess the associations between social, demographic, and economic factors and mental health indicators during the COVID-19 pandemic in Spain. Snowball technique and convenience sampling were followed to recruit participants as follows: (1) Students enrolled in the nursing degree at the Autonomous University of Madrid were contacted by email and through academic platforms. All potential participants contacted were invited to share the study information with other people within their environment. (2) Professors and researchers directly involved in the present research informed their personal and professional contacts of the study by email and invited them to participate and disseminate the information. (3) Social networks (Facebook, Instagram, Twitter) were used to recruit additional participants. The advert of the study was published on behalf of participation by the European University of Madrid which was accessible to the general public. Similarly, the proposal to participate in the study was published in the professional and personal profiles of each of the researchers involved in the present research. Participants as well as those who decided not to participate in the study were able to share the information of the study with their social and professional networks.

After potential participants were informed of the objectives and relevant information of the study, they could indicate consent to participate in the study or not. Upon a positive response, the anonymous questionnaire was deployed. All participants who met the inclusion criteria were recruited. Inclusion criteria were to currently reside in Spain, be aged 18 or over, be able to read, understand, and complete the questionnaire in Spanish, be interested in participating in the study, and provide informed and written consent. Data was collected between March 19th and April 26th, 2020, the most critical periods of the COVID-19 pandemic registered in Spain.

A total of 37 participants were excluded from the study because they did not meet the inclusion criteria of age (they were under 18 years old). Further, participants in the study were asked if they were active health professionals. Those who met this condition (117 participants) were not included in the present analysis, since their status as health workers has important implications for both risk of infection and mental health and well-being status. As a result, 801 participants provided valid data of mental health indicators and were considered for the analysis. The sample size was calculated using the G-Power tool, for a linear multiple regression, considering an Alpha error of 0.05 and a 0.95 statistical power. *Post hoc* statistical power calculations were also carried out, for an alpha error of 0.05 and according to the effect size range obtained in the models (considering the two predictors used), showing a statistical power higher than 0.95 in all cases.

### Measurement Instruments

#### Mental Health Indicators

Three mental health and well-being indicators were considered in the present study: psychological health status and positive and negative affect.

The Goldberg General Health Questionnaire (GHQ-12) was used to assess mental health, employing its short version. This questionnaire is a widely used instrument designed to discriminate whether or not psychological morbidity is present. The validation study of the Spanish version revealed an adequate internal consistency, which ranges between 0.82 and 0.90, a sensitivity between 76 and 100, and a Cronbach’s alpha of 0.76 (Muñoz et al., 1993). The score ranges from 12 to 48, with higher scores indicating worse mental health. In the present sample, a Cronbach’s alpha of 0.849 was found for this scale. An example item of the questionnaire would be “*Have you been able to concentrate on whatever you are doing*?”

The Positive and Negative Affect Schedule (PANAS) was employed to measure well-being. This questionnaire is formed by two independent scales, each consisting of 10 items. The positive affect scale measures feelings such as joy or pleasure, and the negative affect scale includes feelings such as anxiety and sadness. Higher scores indicate higher levels of positive and negative affect. The instrument consists of a Likert scale that ranges from very slightly or not at all, to extremely (Watson et al., 1988). This questionnaire is a widely used instrument to assess positive and negative affect (Linley et al., 2009). In the present study, the Spanish version was used, which respects the same bidimensional structure and shows adequate test–retest reliability (range from 0.79 to 0.93 in both scales) (Ostir et al., 2005) and convergent and discriminant validity (Ortuño-Sierra et al., 2015). The score ranges from 10 to 30 in both scales. In the present sample, a Cronbach’s alpha of 0.715 was found for positive affect, and 0.811 for negative affect. An example item of the scale would be “*Indicate the extent to which you have felt distressed over the past week*.”

#### Social, Demographic, and Economic Factors

Age, number of children, and dwelling size (m^2^) were reported as a number by the participants.

Gender was indicated by asking: what is your gender? (Possible answers were female, male, and other).

Country of origin was indicated after the question: what is your country of origin? (Spain/other).

Level of education was identified by participants as basic level of studies (primary and secondary school), medium level of studies (baccalaureate and technical education), and high level of studies (completed university studies).

Marital status was identified by each participant from the possible answers: married, single, unmarried partner, separated/divorced, and widowed.

Current employment status, during the COVID-19 pandemic was defined as self-employment, employment, unemployment, homemaker, retired, or student.

Living with dependent seniors, current reduced income due to the COVID-19 pandemic, and working outside home (required to continue working as essential workers during the pandemic) were indicated as yes or no.

Length of confinement was calculated from the date of completion of the questionnaire, considering March 14th as the first day of confinement (coinciding with the declaration of state of alarm in Spanish territory).

### Covariates

Self-referred current medical diagnosis of COVID-19 (yes/no) was included as covariate for the analysis, given its potential influence on mental health indicators.

### Ethical Procedures

The protocol for the present study obtained approval from the Ethics Committee of the Faculty of Biomedical and Health Science of the European University of Madrid (No CIPI/20/135). All participants were informed of the purpose and intent of the study and provided written consent. Similarly, anonymity of each of the participants was ensured.

### Data Analyses

All statistical analysis was conducted using the Statistical Package for the Social Sciences software version 21.0 (SPSS. Inc., Chicago, United States) and STATA/SE 14.1 software (Stata Corp LP).

Descriptive statistics (mean values and standard deviations or numbers and percentages) were calculated to describe participant characteristics. Differences between categorical variables and mental health indicators were addressed using Student’s t test for dichotomous variables and ANOVA test for variables with more than 2 categories. The Spearman correlation test was employed to value associations between quantitative variables and mental health indicators after assessing the distribution of each variable using the Kolmogorov–Smirnov test (all, *p* < 0.001).

Linear regression was used to test the association between social, demographic, and economic factors, and mental health indicators. Non-parametric variables were transformed to address normality. Unadjusted models and models adjusted for current medical diagnosis of COVID-19 were fitted. There were no relevant differences between unadjusted and adjusted models; thus, only adjusted models will be shown in the results section.

## Results

Mean and standard deviation (SD) values of the mental health indicators are presented in Table 1. A mean of 25.7 (5.5 SD) for the mental health score, a mean of 24.4 (2.8 SD) for positive affect, and a mean of 18.0 (3.6 SD) for the negative affect score were obtained.

**TABLE 1:** Characteristics of the participants examined.

***n***	**801**	**Mental health score**	***P*^a^**	**Positive affect score**	***P*^b^**	**Negative affect score**	***P*^c^**
Mental health scores (12–48) [mean, SD]	25.7 (5.5)						
Positive affect scores (10–30) [mean, SD]	24.4 (2.8)						
Negative affect scores (10–30) [mean, SD]	18.0 (3.6)						
Current Medical diagnosis COVID-19 infection			0.873^#^		0.893^#^		0.924^#^
Yes	2.9	25.9 (3.9)		24.4 (2.8)		18.0 (2.2)	
No	97.1	25.7 (5.5)		24.3 (2.8)		18.0 (3.7)	
Age [mean, SD]	40.8 (13.8)		<0.001 (−0.23)^x^		0.564 (0.02)^x^		**<0.001 (−0.17)**^x^
Gender (%)			**<0.001**^#^		**<0.001**^#^		**<0.001**^#^
Female	71.0	5.8 (0.2)		24.6 (2.6)		18.5 (3.5)	
Male	29.0	4.3 (0.2)		23.7 (3.0)		16.7 (3.5)	
Other	0.0	–		–		–	
Country of origin (%)			0.701^#^		0.195^#^		0.138^#^
Spain	90.0	5.5 (0.2)		24.3 (2.8)		18.1 (3.6)	
Other	10.0	5.2 (0.5)		24.7 (2.9)		17.4 (3.7)	
Level of education (%)			0.002*		<0.001*		0.008*
Basic level of studies	5.7	25.1 (6.3)		23.6 (2.7)		18.9 (3.8)	
Medium level of studies	33.3	26.8 (5.9)		23.7 (2.9)		18.3 (3.6)	
High level of studies	61.0	25.2 (5.0)		24.8 (2.6)		17.7 (3.6)	
Number of children [mean, SD]	0.8 (1.0)		**<0.001 (−0.15)**^x^		0.912 (0.04)^x^		**0.007 (−0.9)**^x^
Marital status			0.001*		0.091*		0.002*
Married		24.8 (4.7)		24.4 (2.5)		17.6 (3.9)	
Single		26.5 (5.8)		24.2 (3.0)		18.5 (3.4)	
Unmarried partner		26.4 (5.6)		24.7 (2.5)		18.2 (3.2)	
Separated/divorced		24.9 (5.7)		25.0 (2.6)		16.6 (3.9)	
Widowed		25.2 (6.1)		23.0 (3.6)		18.5 (3.6)	
Living with dependent seniors (%)			0.510^#^		**0.006**^#^		**0.006**^#^
Yes	9.0	26.1 (6.0)		23.5 (2.8)		19.1 (3.4)	
No	91.0	25.6 (5.4)		24.4 (2.7)		17.9 (3.6)	
Employment status (%)			<0.001*		<0.001*		0.003*
Self-employment	8.5	24.2 (5.2)		25.2 (2.6)		17.7 (3.9)	
Employment	59.9	25.4 (5.2)		24.7 (2.5)		17.9 (3.6)	
Unemployment	8.1	27.1 (5.5)		23.4 (2.8)		18.5 (3.3)	
Homemaker	2.4	23.9 (5.7)		23.3 (3.4)		19.6 (3.8)	
Retired	7.6	23.8 (4.0)		23.4 (2.8)		16.6 (3.6)	
Student	13.5	28.3 (6.4)		23.5 (3.2)		18.7 (3.5)	
Dwelling size (m^2^) [mean, SD]	114.3 (102.0)		0.444 (−0.02)^x^		0.715 (0.01)^x^		0.468 (−0.26)^x^
Length of confinement [mean, SD]	20.5 (6.5)		0.465 (0.02)^x^		**0.039 (−0.07)**^x^		0.621 (0.17)^x^
Reduced income (%)	26.6		0.543^#^		0.272^#^		0.569^#^
Yes		25.5 (5.6)		24.5 (2.8)		18.1 (3.5)	
No		25.8 (5.4)		24.3 (2.7)		17.9 (3.7)	
Work outside home (%)	26.9		0.245^#^		**0.012**^#^		0.159^#^
Yes		26.1 (5.3)		24.7 (2.6)		18.3 (3.8)	
No		25.6 (5.5)		24.1 (2.8)		17.9 (3.6)	

P^*a*^-value for comparing socioeconomic and labor indicators and mental health score. P^*b*^-value for comparing socioeconomic and labor indicators and positive affect score. P^*c*^-value for comparing socioeconomic and labor indicators and negative affect score. ^*x*^Spearman correlation test, P (correlation coefficient). ^#^T-Student test. *ANOVA test. Bold values mean that p ≤ 0.05.

Characteristics of the participants are also presented in Table 1. Of the participants examined, 2.9% had a current medical diagnosis of COVID-19, a condition which was not associated with mental health indicators. The mental health score was higher in younger people (*p* < 0.001, *r* = −0.23), women (5.8 ± 0.2, *p* < 0.001), people with a medium level of studies (26.8 ± 5.9, *p* = 0.002), those with a lower number of children (*p* < 0.001, *r* = −0.15), single people (26.5 ± 5.8, *p* = 0.001), and students (28.3 ± 6.4, *p* < 0.001). The positive affect score was higher in women (24.6 ± 2.6, *p* < 0.001), people with a high level of studies (24.8 ± 2.6, *p* < 0.001), those not living with dependent seniors (24.4 ± 2.7, *p* = 0.006), self-employees (25.2 ± 2.6, *p* < 0.001), those with a shorter length of confinement (*p* = 0.039, *r* = −0.07), and those working outside home (24.7 ± 2.6, *p* = 0.012). Finally, the negative affect score was higher in younger people (*p* < 0.001, *r* = −0.17), women (18.5 ± 3.5, *p* < 0.001), people with a basic level of studies (18.9 ± 3.8, *p* = 0.008), those with a lower number of children (*p* = 0.007, *r* = −0.9), single and widowed people (18.5 ± 3.4 and 18.5 ± 3.6, respectively, *p* = 0.002), people living with dependent seniors (19.1 ± 3.4, *p* = 0.006), and homemakers (19.6 ± 3.8, *p* = 0.003).

Linear regression models for the mental health score are presented in Table 2. A one-unit increase in age (β = −0.09, 0.01(SE), *p* < 0.001) and in number of children (β = −0.83, 0.18(SE), *p* < 0.001) was associated with decreased mental health scores. Similarly, a high level of studies (β = −1.36, 0.39(SE), *p* = 0.001), being married (β = −1.48, 0.39(SE), *p* < 0.001), being self-employed (β = −1.56, 0.69(SE), *p* = 0.025), and being retired (β = −2.08, 0.73(SE), *p* = 0.004) were linked to lower mental health scores. On the other hand, referring gender as female (β = 1.70, 0.42(SE), *p* < 0.001), reporting a medium level of studies (β = 1.61, 0.40(SE), *p* < 0.001), being single (β = 1.41, 0.39(SE), *p* < 0.001), being unemployed (β = 1.55, 0.71(SE), *p* = 0.029), and being a student (β = 3.04, 0.56(SE), *p* < 0.001) were associated with a higher mental health score.

**TABLE 2:** Linear regression models for *mental health score* (*n* = 801).

			**Model 1**	
	***n***	**β (SE)**	**95% CI**	***P***
Age	801	−0.09(0.01)	−0.12–0.06	**<0.001**
Gender	800			
Women		1.70(0.42)	0.87–2.54	**<0.001**
Country of origin	801			
Other than Spain		−0.22(0.64)	−1.50–1.04	0.727
Level of education	801			
Basic level of studies		−0.66(0.84)	−2.31–0.99	0.434
Medium level of studies		1.61(0.40)	0.80–2.41	**<0.001**
High level of studies		−1.36(0.39)	−2.13–0.58	**0.001**
Number of children	800	−0.83(0.18)	−1.20–0.48	**<0.001**
Marital status	800			
Married		−1.48(0.39)	−2.25–0.70	**<0.001**
Single		1.41(0.39)	0.64–2.18	**<0.001**
Unmarried partner		0.78(0.66)	−0.51–2.08	0.238
Separated/divorced		−0.81(0.75)	−2.28–0.65	0.277
Widowed		−0.47(1.60)	−3.62–2.66	0.767
Living with dependent seniors	801	0.45(0.68)	−0.88–1.78	0.508
Employment status	801			
Self-employment		−1.56(0.69)	−2.93–0.20	**0.025**
Employment		−0.67(0.39)	−1.45–0.10	0.091
Unemployment		1.55(0.71)	0.16–2.95	**0.029**
Homemaker		−1.83(1.27)	−4.34–0.67	0.152
Retired		−2.08(0.73)	−3.52–0.65	**0.004**
Student		3.04(0.56)	1.94–4.14	**<0.001**
Dwelling size (m^2^)	786	−0.00(0.00)	−0.00–0.00	0.178
Length of confinement	801	0.05(0.02)	−2.42–2.14	0.074
Reduced income	801	−0.26(0.44)	−1.13–0.59	0.546
Work outside home	746	0.52(0.45)	−0.36–1.42	0.247

*Statically significant values are in bold. Model 1: Analyses were adjusted for current medical diagnoses COVID-19. β, unstandardized coefficient.*

Linear regression models for positive affect scores are presented in Table 3. Medium level of studies (β = −1.02, 0.20(SE), *p* < 0.001), living with dependent seniors (β = −0.95, 0.34(SE), *p* = 0.006), being unemployed (β = −0.98, 0.36(SE), *p* = 0.007), being retired (β = −1.05, 0.37(SE), *p* = 0.005), and being a student (β = −1.03, 0.28(SE), *p* < 0.001) were linked to decreased positive affect scores. On the other hand, referring gender as female (β = 0.93, 0.21(SE), *p* < 0.001), a high level of studies (β = 1.12, 0.20(SE), *p* < 0.001), being self-employed (β = 0.94, 0.35(SE), *p* = 0.008), being employed (β = 0.92, 0.20(SE), *p* < 0.001), and working outside home (β = 0.59, 0.23(SE), *p* = 0.012) were linked to a higher positive affect score.

**TABLE 3:** Linear regression models for *positive affect score* (*n* = 801).

			**Model 1**	
	***n***	**β (SE)**	**95% CI**	***P***
Age	801	0.00(0.00)	−0.00–0.01	0.517
Gender	800			
Women		0.93(0.21)	0.51–1.36	**<0.001**
Country of origin	801			
Other than Spain		0.43(0.33)	−0.22–1.08	0.194
Level of education	801			
Basic level of studies		−0.73(0.43)	−1.58–0.11	0.090
Medium level of studies		−1.02(0.20)	−1.43–0.62	**<0.001**
High level of studies		1.12(0.20)	0.73–1.51	**<0.001**
Number of children	800	0.05(0.09)	−0.13–0.24	0.564
Marital status	800			
Married		0.07(0.20)	−0.32–0.47	0.713
Single		−0.29(0.20)	−0.68–0.10	0.147
Unmarried partner		0.34(0.34)	−0.32–1.01	0.312
Separated/divorced		0.66(0.38)	−0.08–1.42	0.082
Widowed		−1.41(0.81)	−3.02–0.18	0.084
Living with dependent seniors	801	−0.95(0.34)	−1.63–0.27	**0.006**
Employment status	801			
Self-employment		0.94(0.35)	0.25–1.64	**0.008**
Employment		0.92(0.20)	0.52–1.31	**<0.001**
Unemployment		−0.98(0.36)	−1.70–0.27	**0.007**
Homemaker		−1.11(0.65)	−2.39–0.16	0.089
Retired		−1.05(0.37)	−1.78–0.32	**0.005**
Student		−1.03(0.28)	−1.59–0.46	**<0.001**
Dwelling size (m^2^)	786	0.00(0.00)	−0.00–0.00	0.186
Length of confinement	801	−0.02(0.01)	−0.05–0.00	0.122
Reduced income	801	0.24(0.22)	−0.19–0.69	0.271
Work outside home	746	0.59(0.23)	0.13–1.05	**0.012**

*Statically significant values are in bold. Model 1: Analyses were adjusted for current medical diagnoses COVID-19. β, unstandardized coefficient.*

Finally, linear regression models for negative affect scores are presented in Table 4. A one-unit increase in age (β = −0.04, 0.00(SE), *p* < 0.001) and in number of children (β = −0.35, 0.12(SE), *p* = 0.004) was associated with lower negative affect scores. Also, a high level of studies (β = −0.77, 0.26(SE), *p* = 0.004), being married (β = −1.58, 0.26(SE), *p* = 0.028), being separated or divorced (β = −1.45, 0.50(SE), *p* = 0.004), being and retired (β = −1.50, 0.48(SE), *p* = 0.002) are linked to lower negative affect scores. However, reporting gender as female (β = 1.85, 0.28(SE), *p* < 0.001), basic studies (β = 1.25, 0.56(SE), *p* = 0.027), being single (β = 0.85, 0.26(SE), *p* = 0.001), living with dependent seniors (β = 1.23, 0.45(SE), *p* = 0.006), being a homemaker (β = 1.68, 0.85(SE), *p* = 0.049), and being a student (β = 0.81, 0.38(SE), *p* = 0.033) were related to higher negative affect scores.

**TABLE 4:** Linear regression models for *negative affect score* (*n* = 801).

			**Model 1**	
	***n***	**β (SE)**	**95% CI**	***P***
Age	801	−0.04 (0.00)	−0.06–0.02	**<0.001**
Gender	800			
Women		1.85 (0.28)	1.30–2.40	**<0.001**
Country of origin	801			
Other than Spain		−0.64 (0.42)	−1.49–0.20	0.138
Level of education	801			
Basic level of studies		1.25 (0.56)	0.14–2.35	**0.027**
Medium level of studies		0.52 (0.27)	−0.01–1.06	0.055
High level of studies		−0.77 (0.26)	−1.29–0.25	**0.004**
Number of children	800	−0.35 (0.12)	−0.60–0.11	**0.004**
Marital status	800			
Married		−1.58 (0.26)	−1.10–0.06	**0.028**
Single		0.85 (0.26)	0.34–1.37	**0.001**
Unmarried partner		0.24 (0.44)	−0.62–1.11	0.582
Separated/divorced		−1.45 (0.50)	−2.43–0.47	**0.004**
Widowed		0.46 (1.07)	−1.63–2.57	0.663
Living with dependent seniors	801	1.23 (0.45)	0.35–2.12	**0.006**
Employment status	801			
Self-employment		−0.33 (0.46)	−1.25–0.58	0.475
Employment		−0.16 (0.26)	−0.69–0.35	0.528
Unemployment		0.51 (0.47)	−0.42–1.44	0.284
Homemaker		1.68 (0.85)	0.00–3.35	**0.049**
Retired		−1.50 (0.48)	−2.46–0.54	**0.002**
Student		0.81 (0.38)	0.06–1.55	**0.033**
Dwelling size (m^2^)	786	−0.00 (0.00)	−0.00–0.00	0.459
Length of confinement	801	−0.01 (0.02)	−0.05–0.02	0.528
Reduced income	801	0.16 (0.29)	−0.41–0.74	0.568
Work outside home	746	0.43 (0.30)	−0.17–1.03	0.161

*Statically significant values are in bold. Model 1: Analyses were adjusted for current medical diagnoses COVID-19. β, unstandardized coefficient.*

## Discussion

According to our results, the most vulnerable populations in terms of mental health morbidity were women, younger people, individuals with a medium level of studies, those with fewer children, single individuals, students, and the unemployed. In contrast, positive affect was higher among women, those with a high level of studies, those not co-living with dependent seniors, the self-employed, the employed, and those working outside home due to the COVID-19 pandemic. Lastly, negative affect was negatively associated with age and number of children and was higher among females, people with a basic level of studies, single individuals and those with unmarried partners, individuals co-living with dependent seniors, homemakers, and students.

As expected, and in line with prior studies, more vulnerable individuals in terms of socioeconomic status were more likely to report symptoms of psychological distress (Blendon et al., 2004; Cava et al., 2005; Reynolds et al., 2008; Taylor et al., 2008; Braunack-Mayer et al., 2013). Educational level and employment status were related to psychological morbidity and well-being. Those with high educational status showed lower psychological morbidity and a more favorable positive affect score, which coincides with prior studies in Australia collected during the influenza pandemic in Taylor et al. (2008), and recent studies in Spain (Domínguez-Salas et al., 2020; Gómez-Salgado et al., 2020; Rodríguez-Rey et al., 2020a). Although the causes are unknown, it may be interpreted that a lower educational level, in itself a good indicator of socioeconomic status (Galobardes et al., 2006), could be linked to higher socioeconomic vulnerability and thus act as a stressor in a situation of uncertainty, worsening people’s psychological discomfort (Zahran et al., 2011). Similar effects are estimated in the case of employment status when explaining higher psychological morbidity among unemployed people. As has been previously found in Spanish studies during the COVID-19 (Gómez-Salgado et al., 2020), unemployment may lead to higher psychological distress (Esteban-Gonzalo et al., 2018), especially in a context of socioeconomic uncertainty. Studies carried out under normal conditions in Europe indicated that unemployment leads to deterioration of health status, especially among women and people in prime working age (Heggebø, 2016). Concretely in Spain, recession periods have contributed to poorer mental health among unemployed men and women, to the point of increasing suicide rates (Córdoba-Doña et al., 2016; Rivera et al., 2016). Those who were active, in terms of being able to keep a remunerated job and at the same time preserve their working routines and incomes, showed higher levels of positive affect. The higher psychological distress found among students is also remarkable and is probably attributable to negative expectations as to their career advancement.

Unexpectedly, no significant associations were found between length of confinement, mental health morbidity, and negative/positive affect. Prior studies have found contradictory results. While some pointed out negative effects of quarantine duration on psychological health (Hawryluck et al., 2004; Robertson et al., 2004), not all studies could assure such effects (Brooks et al., 2020). The progress and evolution of the outbreak in the Spanish case should be contextualized. COVID-19 impacted the Spanish territory very rapidly during the first weeks, collapsing the sanitary system and generating panic in the population. The first period of quarantine was especially dramatic given the amount of negative news in the media informing of the progress of the pandemic, characterized by an increasing number of deaths. Thus, uncertainty, fear and hysteria were dominant feelings during this first period (Tapia and Jerónimo, 2020; Zaar and Ávila, 2020). An improvement in this critical situation during the second period of the crisis may have contributed to balance the malaise of the population. Similar findings have been observed by other Spanish researchers. Specifically, a study carried out during the first 3 weeks of confinement found that the odds of having a higher level of health risk behaviors (a change toward a higher number of health risk behaviors than before the confinement) decreased during the confinement, suggesting that the Spanish adult population may have adapted to the new situational context by gradually improving their health behaviors (López-Bueno et al., 2020a). For instance, the same researchers found significant inverse associations between overall adherence to physical activity and current perceived anxiety, proposing that higher levels of perceived anxiety and worse mood might be mitigated by a minimum amount of weekly physical activity, which increased in the confinement context (López-Bueno et al., 2020b).

Stressors during quarantine should also be considered. Number of children, co-living with a dependent senior, and being alone were found to be related to mental health or well-being. It was also unexpected to find that an increased number of children at home was associated with better mental health status and lower levels of negative affect. Prior studies have identified similar tendencies, suggesting the protective effects of having two or more children at home (Taylor et al., 2008). However, having only one child may, paradoxically, be counterproductive in terms of mental health (Taylor et al., 2008). In a context in which children are deprived of social interactions, one might hypothesize that co-living with other children could compensate the lack of social stimulus. Children with siblings could maybe enjoy a game companion at home, substituting other friends and colleagues and minimizing the impact of confinement. This fact could improve both children’s and parents’ well-being in terms of delegating more responsibility to older children.

Dependent seniors co-living at home were found to be a stressor during quarantine, with lower levels of positive affect and higher levels of negative affect among caregivers, a finding validated by the existing literature on caregiving and its damaging effects on mental health and well-being (Shifren and Kachorek, 2003; MacNeil et al., 2010). This aspect may be particularly salient in the context of the quarantine in which external support is lacking.

Lastly, although previous literature suggests that having more space at home might be related to increased well-being (Ratcliffe, 2010; Nakazato et al., 2011; Solari and Mare, 2012), particularly in a context of confinement in which movements outside the home are restricted, no associations between dwelling size and mental health indicators were observed in the present study. Some studies carried out in normal conditions have found that dwelling conditions may affect psychological well-being albeit indirectly, in that the relation is due more to the extent to which a person’s expectations of residential satisfaction are met (Phillips et al., 2005). Other studies have stated that financial capability may be a significant moderator between dwelling size and well-being (Taylor et al., 2011).

Finally, sociodemographic factors should also be considered. Age, gender, and marital status have been found to be related to mental health and well-being. In congruence with prior studies carried out in Spain during the COVID-19, women showed higher mental morbidity (Taylor et al., 2008; Domínguez-Salas et al., 2020; Rodríguez-Rey et al., 2020a) and higher scores in positive and negative affect. The fact that women usually experience lower levels of mental health and well-being is a well-known phenomenon (Seedat et al., 2009; Heise et al., 2019), with higher emotional intensity and higher levels in both positive and negative affect (Thomsen et al., 2005; Burns and Machin, 2010). However, quarantine may entail a multiplier effect if the amount of responsibilities at home, specifically for women with children, are taken into account (Taylor et al., 2008). Similar effects have been identified with respect to age, with better mental health and well-being in older individuals. Previous and recent studies carried out during the COVID-19 outbreak in Spain and other European countries have also found a protective effect of age in quarantine contexts, suggesting that younger people are particularly vulnerable, do not cope as well with the situation, and are also less likely to be resilient when it comes to coping with adversity (Taylor et al., 2008; Bruine de Bruin, 2020; Rodríguez-Rey et al., 2020a; Skoog, 2020). Also, one may hypothesize that older adults are better trained in practicing self-control and resilience. Some studies have found that self-control interacts with age, enhancing perceived control by older individuals, at least in normal contexts (Sinha et al., 2002). Older adults are capable of high resilience despite socioeconomic backgrounds, personal experiences, and declining health (MacLeod et al., 2016).

This study is not without its limitations, as follows: (1) Perhaps the most relevant limitation we must point out is that the use of social networks to recruit participants for this study may associate a sample selection bias. However, the need to assume this limitation was due to the confinement of the entire Spanish population for the full duration of data collection. There were scarce possibilities of reaching potential participants by other means. In spite of the limitations associated with the use of social networks for data collection, the decision to proceed was supported by some scientific works that have pointed out that social media data maintain the capacity for addressing broad social questions while upholding methodological integrity (Davis and Love, 2019). Other studies carried out in the same temporal and geographical context have also assumed this remarkable but insurmountable limitation (Rodríguez-Rey et al., 2020a,b). Therefore, our results must be considered with caution, since they will not be generalizable to the general Spanish population. (2) The cross-sectional design of the studio does not allow us to establish cause-and-effect relationships. We can only report associations between mental health indicators and social, demographic, and economic factors. Future longitudinal studies should be carried out to extend the cross-sectional perspective examined in this study. (3) Although all questionnaires were carefully selected and all are valid and reliable, the variables are self-reported, which could bias the inherent quality of the data. (4) Unmeasured covariates or the presence of measurement error in the covariates included in the models may lead to residual confounding. In addition, we lack information related to the psychiatric history of the participants, teleworking, infection or death of a close relative, children’s age, and number of hours consulting information on COVID, which may influence mental health status and well-being during confinement. (5) Information regarding geographical area of residence was not available, which may play a role since the territory was not equally affected by COVID-19. However, the unifying factor of the national state of alarm and the confinement of the population throughout Spain must be considered, with its repercussions at the psychological level regardless of the rate of infection. For this reason, the regression models were controlled by the medical diagnosis of Covid reported by participants. Regardless of the Covid infection rate in each area, all regions of Spain were confined under the same restrictions during the data collection period (Real Decreto 465/2020, de 17 de marzo). (6) Finally, one of the most vulnerable social groups in the context in which this work was developed is the older adult population. Although a total of 103 participants reported to be 60 years old or older, unfortunately we cannot offer a specific vision of the problem in terms of mental health of this sector of the population. Future studies should be specifically directed at understanding the mental health conditions of this group and associated factors.

However, this study provides information about social, demographic, and economic factors able to influence the mental health of a population unable to exert their basic freedoms in the unique instance of a health emergency.

In conclusion, the most vulnerable populations in terms of mental health morbidity and well-being were women, younger people, people with basic or medium level of studies, students and individuals with no remunerated activities, singles, and those with unmarried partners. Stressors during confinement were co-living with dependent seniors and having few children. These results highlight the need to consider psychosocial predictors of mental health and well-being in order to design and implement future intervention programs to monitor mental health and well-being outcomes among the most vulnerable individuals in the highly probable context of future pandemics.

## Data Availability Statement

The raw data supporting the conclusions of this article will be made available by the authors, under limited conditions.

## Ethics Statement

The protocol for the present study obtained approval from the Ethics Committee of the Faculty of Biomedical and Health Science of the European University of Madrid. The patients/participants provided their written informed consent to participate in this study.

## Author Contributions

SE-G was the coordinator, main investigator of the study, and oriented and revised the article. LE-G was the coordinator and main investigator of the study, and analyzed the data. JG-P was an investigator of the study and revised the article. MC-G was an investigator of the study and revised the article. All authors have read and agreed to the published version of the manuscript.

## Conflict of Interest

The authors declare that the research was conducted in the absence of any commercial or financial relationships that could be construed as a potential conflict of interest.
